# SARS-CoV-2 infection in London, England: changes to community point prevalence around lockdown time, March–May 2020

**DOI:** 10.1136/jech-2020-214730

**Published:** 2020-10-01

**Authors:** Michael Edelstein, Chinelo Obi, Meera Chand, Susan Hopkins, Kevin Brown, Mary Ramsay

**Affiliations:** National Infection Service, Public Health England, London, UK

**Keywords:** Epidemics, communicable diseases, control of diseases

## Abstract

**Background:**

The UK has been one of the European countries most affected by COVID-19 pandemic. The UK implemented a lockdown in March 2020, when testing policy at the time was focusing on hospitalised cases. Limited information is therefore available on the impact of the lockdown on point prevalence in the community. We assessed COVID-19 point prevalence in London between early April and early May 2020, which approximately reflect infection around the time of the lockdown and 3–5 weeks into lockdown.

**Methods:**

We tested 1064 participants of a community surveillance cohort for acute COVID-19 infection using PCR in London in April and May 2020 and described positivity as well as characteristics and symptoms of the participants.

**Results:**

Point prevalence decreased from 2.2% (95% CI 1.4 to 3.5) in early April to 0.2% (95% CI 0.03 to 1.6) in early May. 22% of those who tested positive in April were asymptomatic. Extrapolation from reports of confirmed cases suggest that 5–7.6% of total infections were confirmed by testing during this period.

**Conclusion:**

COVID-19 point prevalence in the community sharply decreased after lockdown was implemented. This study is based on a small sample and regular seroprevalence studies are needed to better characterise population-level immunity.

## INTRODUCTION

The WHO declared COVID-19, a respiratory disease caused by the severe acute respiratory syndrome coronavirus 2 (SARS-CoV-2), a pandemic on March 11, 2020. The UK reported its first case on January 31, and as of May 18, 2020, 243 695 confirmed cases had been reported.^1^ At the beginning of the study in early April, UK policy was to prioritise available tests for patients requiring hospitalisation and outbreaks in institutional settings. Evidence suggests that over 80% of COVID-19 cases are mild^2^ and that the proportion who are asymptomatic when tested may be as high as 80%.^3^ Therefore, using laboratory confirmation in hospitalised cases will severely underestimate disease incidence. Repeated testing among individuals in the community will help estimate trends in COVID-19 point prevalence and help to describe the characteristics of milder infection. On March 23, a national lockdown was implemented to control the spread of COVID-19 in the UK. It ordered UK citizens to stay in their homes except for very limited purposes. We assessed COVID-19 point prevalence in London between early April and early May 2020, which approximately reflect infection around the time of the lockdown, and 3–5 weeks into lockdown.

## METHODS

Flusurvey (Flusurvey.net) is a participative surveillance cohort set up for influenza surveillance^4^ and adapted for COVID-19. Any member of the UK public can register and report on symptoms weekly. On March 27, 2020, all Flusurvey participants resident in London were asked for their consent and the consent of their household members, to receive a nasal swab for COVID-19 testing. Self-swabbing has previously been shown to be acceptable to Flusurvey participants.^5^ Swabs can be stored at room temperature and use packaging compliant with UN3733^6^ posted through regular mail. Together with the swab, Flusurvey participants and their household members were asked to report any cough, fever, shortness of breath and loss of smell in the 2 weeks prior to the swab using a short questionnaire. The first set of swabs and questionnaires were posted to participants on March 31, 2020 (wave 1). In the second phase of swabbing, half of the wave 1 participants were randomly selected and sent a swab between April 29, 2020 and April 30, 2020 (wave 2). In order to ascertain prelockdown point prevalence in the first wave of sampling, we divided wave 1 samples between timely (collected before April 7, 2020) and late (collected April 7, 2020 and April 28, 2020) samples.

Nasal swabs were tested for viral RNA as soon as practical after reception, usingPCR at the Public Health England Respiratory Virus Unit using primers targeted to either the RdRp and/or Orf gene.^7 8^ Participants who tested positive were informed of their result.

The age and sex distribution of the initial respondents were compared with the London population,^9^ and positivity rate by sex and age group calculated, together with 95% CIs crude and age-adjusted using direct standardisation with London population as standard population. We described the symptoms that timely wave 1 participants reported and their association with being a case, using prevalence ratios of specific symptoms together with 95% CIs. We calculated the positive predictive value of each of the reported symptoms as well as for fever and cough as a cluster. We calculated the crude and age-adjusted positivity rate among participants who returned their first swab late and their second swab, with 95% CIs.

## RESULTS

One thousand three hundred and seventy-three individuals (582 London Flusurvey participants and 791 household members) consented to self-swabbing. By May 12, 2020, 1081 first wave samples were received (response rate: 79%) of which 17 were excluded because the participant no longer reported a London address. The participants who sent the remaining 1064 samples were distributed across all of London boroughs, although the sample under-represented children (13.7% under 19 in FluSurvey vs 24.8% in London) and over-represented older adults (27.9% in Flusurvey vs 21.5% in London). Age distribution of participants did not differ significantly by sex (p=0.8). Of the 698 individuals who received a second swab, 444 had returned them by May 12, 2020 when follow-up ended (response rate 63.6%). Of the 1064 wave 1 samples received, 816 were timely and 248 were late.

Of the 816 timely samples from the first wave, 18 were positive in 14 households (crude positivity 2.2%, age-adjusted 2.3%, 95% CI 1.2 to 3.4). Positivity was higher among women and among 10-year-olds to 19-year-olds (table 1). Of the 18 positive individuals, 14 (78%) had experienced symptoms in the last 2 weeks and 4 (22%) had not. Symptom onset among symptomatic participants ranged from March 15 to March 30 and time from symptom onset to sample collection ranged from 4 to 19 days. Cough was the most commonly reported symptom among cases and all symptoms were significantly associated with being COVID-19-positive (table 2). The positive predictive value of these symptoms ranged from 7.1% to 13.5% (table 2). Of the 248 late wave 1 samples, two (both women and symptomatic) were positive (crude positivity 0.8%, age-adjusted 0.7%, 95% CI 0 to 1.6), and only 1 of 444 samples received from wave 2 was positive (crude positivity 0.2%, age-adjusted 0.2%, 95% CI 0 to 0.7). The case was a women, asymptomatic, not a household contact of a previously identified case and had tested negative in early April. Of the 20 positive participants in phase 1 of swabbing, 5 (including 3 asymptomatic) were retested and all were negative. Of the three asymptomatic cases who were retested, none reported symptoms when asked again. Thirty-seven individuals lived in the 16 households which had a least one case. Household size ranged from 1 to 4 individuals. Five households had two cases. No additional cases were found during wave 2 in the five households that were tested in both waves and had an individual who tested positive in wave 1.

**Table 1:** COVID-19 positivity rate by testing period, age and sex

	Participants	Sex		Age, years					
		Men	Women	<10	10–19	20–44	45–54	55–64	65+
Samples collected before April 7, 2020 (timely wave 1)	COVID-19 positive (n)	5	13	1	3	9	1	3	1
	COVID-19 negative (n)	395	403	58	56	336	118	123	107
	Positivity (%, 95% CI)	1.2 (0.4 to 2.9)	3.1 (1.7 to 5.3)	1.7 (0.04 to 9.1)	5.1 (1.1 to 14.1)	2.6 (1.2 to 4.9)	0.8 (0.02 to 4.6)	2.4 (0.5 to 6.8)	0.9 (0.02 to 5)
Samples collected from April 7 to April 28, 2020 (late wave 1)	COVID-19-positive (n)	0	2	0	0	1	0	0	1
	COVID-19-negative (n)	106	140	12	16	114	44	25	35
	Positivity (%, 95% CI)	–	1.4 (0.3 to 5.5)	–	–	0.9 (0.1 to 6)	–	–	2.8 (0.4 to 18.3)
Collected on April 29 or after (wave 2)	COVID-19-positive (n)	0	1	0	0	1	0	0	0
	COVID-19-negative (n)	214	229	35	40	165	74	69	60
	Positivity (%, 95% CI)	–	0.4 (0.06 to 3.1)	–	–	0.6 (0.08 to 4.2)	–	–	–

**Table 2:** Distribution of symptoms among wave 1 Flusurvey London participants

Symptom	Number of COVID-19-positivereporting (n=18)		Number of COVID-19-negativereporting (n-=798)		Prevalence ratio	95% CI	Positive predictive value (%)
	n	%	n	%			
Fever	7	39	45	6	6.9	3.6 to 13.1	13.5
Cough	8	44	92	12	3.8	2.2 to 6.7	8
Fever or cough	11	61	101	13	4.8	3.27 to.3	9.8
Shortness of breath	5	28	45	6	4.9	2.2 to 10.9	10
Loss of smell	5	28	46	6	4.8	2.2 to 10.7	8

## DISCUSSION

This study is the first to describe changes in the point prevalence of COVID-19 infection in the community in London, and England more generally. The 2.3% point prevalence reflects infections acquired in London shortly before and after the lockdown on March 23, 2020, and the positivity rate of 0.2% reflects infections during the second half of April, 3–5 weeks into the lockdown. The observational data collected and described here cannot establish causality and merely describes point prevalence around the time of the lockdown. Nevertheless, the lockdown is the most plausible explanation for the observed changes and the decrease in incidence suggests that the lockdown was effective at reducing COVID-19 transmission in London. Positivity is likely to be an underestimate; apart from the usual limitations of PCR diagnostics, self-testing may be slightly less sensitive than administered swabs.^10^

Around the time of our second swab, a pilot survey across England found a similar infection prevalence of 0.27% in early May.^11^ In Iceland, a PCR-based screening in a random sample of the population had a 0.6% positivity.^12^ In Vo, a town in the Veneto region of Italy, screening of the entire population around the time of the lockdown showed a 2.6% positivity.^13^ The London point prevalence in early April was expected to be higher than in other UK regions, based on the number of hospitalised cases per population at the time of swabbing.^1^

Evidence suggests that, in mild cases, the virus is detectable in the upper respiratory tract by PCR from approximately 2 days before to 10 days after the onset of symptoms in over 90% of cases.^14 15^ A further 10% are positive at 14 days, with occasional reports of longer-term detection.^14^ Based on these values, a community PCR point prevalence survey, which only identifies acutely infected individuals, is broadly equivalent to the number of cases acquired over a 12-day period plus an additional 10% of cases acquired in the 4 days before that. Assuming a 5-day lag between disease onset and hospital admission (and testing), and using April 3 as the most common date for the first set of swabs, our infections should correspond to those presenting to hospital and getting tested between March 23 and April 7. Over that time, 10 007 cases were reported in London,^1^ corresponding to a point prevalence of 0.11%. Compared with the 2.3% positivity from our survey, this suggests that, in London, only 4.8% of cases were being confirmed through the testing strategy at that time. During the time period around the second swabbing (April 23 to May 8, determined using a similar approach) there were 2254 reported cases in London, a point prevalence of 0.025% suggesting that 7.6% of infections were detected. These crude figures should be interpreted with caution because viral shedding likely declines over time, affecting test sensitivity in a more complex way. Nevertheless, this figure is consistent with a model that estimated that between 4% and 8.4% of symptomatic cases were being confirmed through testing in hospitals.^16^

Two findings were unexpected. First, the positivity was higher among women than among men, whereas sex-disaggregated data for COVID-19 show equal numbers of cases between men and women.^17^ This could be due to a higher proportion of mild cases among women as higher severity among men is well documented.^18^ Second, 45% of the cases with a date of symptom onset tested positive more than 10 days after the beginning of their symptoms; this compares published data suggesting 90% of mild cases testing negative 10 days postsymptom onset.^14^ These findings warrant further investigation.

The repeated questionnaire and swabbing enabled us to confirm that all retested positive cases who reported no symptoms were genuinely asymptomatic rather than presymptomatic.

There are limitations to our data. First, the sample is relatively small and purposive (as individuals self-select to register with the cohort) and is therefore likely to not be representative on characteristics beyond age and sex. Second, limited information about timing and symptoms makes reconstructing timelines among households difficult. Third, the response rate was lower in the second swabbing. The reason for the decrease is not known. There are several possibilities including ‘testing fatigue’ and less time to complete the second swabbing. As a result, the precision of the second positivity rate is lower and the CIs are wide. Fourth, we made a number of assumptions when estimating underascertainment and did not fully take into account the temporal distribution of likelihood of PCR detection. Linking the swabbing results with information collected weekly in Flusurvey may enable us to reconstruct timelines for each case and to document the duration of symptoms and risk factors associated with becoming infected. This data should be interpreted in conjunction with seroprevalence studies, which are ongoing.

**What is already known on this subject**

- The UK is one of the European countries most affected by COVID-19.
- An initial policy of testing hospitalised cases only means disease incidence is underestimated.
- Implementing a lockdown was followed by a decrease in reported (hospitalised) cases, but its impact on community point prevalence is unknown.

**What this study adds**

- Community point prevalence in London was 2.2% at the time of the lockdown and decreased to 0.2% 3–5 weeks later.
- The proportion of asymptomatic cases in the community was estimated at 22%.
- Reported cases during this period were estimated at 4.8–7.6% of the total number of infections in London.

## References

[1] Public health England and NHSX Coronavirus (COVID-19) in the UK. Available https://coronavirus.data.gov.uk/

[2] The Novel Coronavirus Pneumonia Emergency Response Epidemiology Team Vital surveillances: the epidemiological characteristics of an outbreak of 2019 novel coronavirus diseases (COVID-19): China, 2020. *China CDC Weekly* 2020;2:113–22. 10.46234/ccdcw2020.032 PMC8392929 34594836

[3] Sutton D, Fuchs K, D’Alton M, et al. Universal screening for SARS-CoV-2 in women admitted for delivery. *N Engl J Med* 2020;382:2163–4. 10.1056/NEJMc2009316 32283004 PMC7175422

[4] Adler AJ, Eames KT, Funk S, et al. Incidence and risk factors for influenza-like-illness in the UK: online surveillance using Flusurvey. *BMC Infect Dis* 2014;1:232 10.1186/1471-2334-14-232 PMC4025540 24885043

[5] Wenham C, Gray ER, Keane CE, et al. Self-swabbing for virological confirmation of influenza-like illness among an internet-based cohort in the UK during the 2014–2015 flu season: pilot study. *J Med Internet Res* 2018;20:e71 10.2196/jmir.9084 PMC5856931 29496658

[6] Public Health England Packaging and transport requirements for patient samples: UN3373. Available https://www.gov.uk/government/publications/packaging-and-transport-requirements-for-patient-samples-un3373

[7] Corman VM, Landt O, Kaiser M, et al. Detection of 2019 novel coronavirus (2019-nCoV) by real-time RT-PCR. *Euro Surveill* 2020;25:3 10.2807/1560-7917.ES.2020.25.3.2000045 PMC6988269 31992387

[8] National Centre for Viral Disease Control and Prevention Specific primers and probes for detection 2019 novel coronavirus. Available http://ivdc.chinacdc.cn/kyjz/202001/t20200121_211337.html

[9] Office for national Statistics National population projections: 2018-based. Available https://www.ons.gov.uk/peoplepopulationandcommunity/populationandmigration/populationprojections/bulletins/nationalpopulationprojections/2018based

[10] Seaman CP, Tran LTT, Cowling BJ, et al. Self-collected compared with professional-collected swabbing in the diagnosis of influenza in symptomatic individuals: a meta-analysis and assessment of validity. *Clin Virol* 2019;118:28–35. 10.1016/j.jcv.2019.07.010 31400670

[11] Office for National Statistics Coronavirus (COVID-19) infection survey pilot: England, 14 may 2020. Available https://www.ons.gov.uk/peoplepopulationandcommunity/healthandsocialcare/conditionsanddiseases/bulletins/coronaviruscovid19infectionsurveypilot/england14may2020

[12] Gudbjartsson DF, Helgason A, Jonsson H, et al. Spread of SARS-CoV-2 in the icelandic population. *N Engl J Med* Epub ahead of print 14 Apr 2020;382:2302–15. 10.1056/NEJMoa2006100 32289214 PMC7175425

[13] Lavezzo E, Franchin E, Ciavarella C Suppression of COVID-19 outbreak in the municipality of Vo, Italy. *medRxiv* Preprint 10.1101/2020.04.17.20053157v1.full.pdf

[14] Liu Y, Yan LM, Wan L, et al. Viral dynamics in mild and severe cases of COVID-19. *Lancet Infect Dis* 2020 Mar 19. pii: S1473-3099(20)30232–2 10.1016/S1473-3099(20)30232-2 PMC7158902 32199493

[15] Tan W, Lu Y, Zhang J, et al. Viral kinetics and antibody responses in patients with COVID-19. 10.1101/2020.03.24.20042382v1

[16] Russell TW, Hellewell J, Abbott S et al. Using a delay-adjusted case fatality ratio to estimate under-reporting. Available https://cmmid.github.io/topics/covid19/global_cfr_estimates.html

[17] Hu Y, Sun J, Dai Z, et al. Prevalence and severity of corona virus disease 2019 (COVID-19): a systematic review and meta-analysis. *J Clin Virol* 2020;14:104371 10.1016/j.jcv.2020.104371 PMC7195434 32315817

[18] Intensive care national audit and research centre ICNARC report on COVID-19 in critical care. May 1st 2020 Available https://www.icnarc.org/DataServices/Attachments/Download/f48efee2-d38b-ea11-9125-00505601089b

